# Airway Hyperresponsiveness in Asthma: Mechanisms, Clinical Significance, and Treatment

**DOI:** 10.3389/fphys.2012.00460

**Published:** 2012-12-10

**Authors:** John D. Brannan, M. Diane Lougheed

**Affiliations:** ^1^Respiratory Function Laboratory, Department of Respiratory and Sleep Medicine, Westmead HospitalSydney, NSW, Australia; ^2^Department of Medicine (Respirology), Queen’s UniversityKingston, ON, Canada; ^3^Department of Biomedical and Molecular Sciences (Physiology), Queen’s UniversityKingston, ON, Canada

**Keywords:** airway hyperresponsiveness, bronchial provocation, asthma, pharmacotherapy, diagnosis

## Abstract

Airway hyperresponsiveness (AHR) and airway inflammation are key pathophysiological features of asthma. Bronchial provocation tests (BPTs) are objective tests for AHR that are clinically useful to aid in the diagnosis of asthma in both adults and children. BPTs can be either “direct” or “indirect,” referring to the mechanism by which a stimulus mediates bronchoconstriction. Direct BPTs refer to the administration of pharmacological agonist (e.g., methacholine or histamine) that act on specific receptors on the airway smooth muscle. Airway inflammation and/or airway remodeling may be key determinants of the response to direct stimuli. Indirect BPTs are those in which the stimulus causes the release of mediators of bronchoconstriction from inflammatory cells (e.g., exercise, allergen, mannitol). Airway sensitivity to indirect stimuli is dependent upon the presence of inflammation (e.g., mast cells, eosinophils), which responds to treatment with inhaled corticosteroids (ICS). Thus, there is a stronger relationship between indices of steroid-sensitive inflammation (e.g., sputum eosinophils, fraction of exhaled nitric oxide) and airway sensitivity to indirect compared to direct stimuli. Regular treatment with ICS does not result in the complete inhibition of responsiveness to direct stimuli. AHR to indirect stimuli identifies individuals that are highly likely to have a clinical improvement with ICS therapy in association with an inhibition of airway sensitivity following weeks to months of treatment with ICS. To comprehend the clinical utility of direct or indirect stimuli in either diagnosis of asthma or monitoring of therapeutic intervention requires an understanding of the underlying pathophysiology of AHR and mechanisms of action of both stimuli.

## Introduction

Airway hyperresponsiveness (AHR) is one of the hallmark features of asthma. Bronchial provocation tests (BPTs) are used to assess the presence of AHR to assist in making a clinical diagnosis of asthma in individuals with symptoms and signs that suggest asthma. Asthma is defined as a chronic inflammatory disorder of the airways in which many cells and cellular elements play a role [Global Initiative for Asthma (GINA), 2007]. The chronic inflammation is associated with AHR that leads to recurrent episodes of wheezing, chest tightness, and coughing [Global Initiative for Asthma (GINA), 2007]. It is also understood that the pathophysiology of asthma can lead to a variety of more permanent changes in the airway, which is commonly known as “remodeling” (Holgate et al., 2010). Asthma symptoms are the most clinically accessible marker of disease activity. However, symptoms often do not reflect the degree of airway inflammation and AHR, the two key features attenuated by the mainstay therapy for asthma, inhaled corticosteroids (ICS; Sont et al., 1996).

Bronchial provocation tests are useful in a clinical setting if spirometry is normal and a reversibility test using a standard dose of β_2_-agonist does not demonstrate significant reversibility or bronchodilatation. They may also be useful if a past history of asthma has not been substantiated by objective measures of lung function documenting reversible airflow obstruction, as well as in the evaluation of atypical symptoms such as chronic cough (Irwin, 2006). BPTs may be particularly relevant to perform prior to beginning an occupation or sporting activity that may exacerbate or cause an attack of asthma, and to diagnose and monitor work-related asthma (Tarlo et al., 2008).

Bronchial provocation tests have played a significant role in research to understand mechanisms of AHR, mechanisms of dyspnea and cough in asthma (Lougheed et al., 1993, 1995; Turcotte and Lougheed, 2011) and to investigate the efficacy of pharmacotherapy used in the prevention of AHR and treatment of asthma (O’Byrne et al., 2009a). BPTs have been used to investigate AHR following both short- and long-term treatment with regular ICS in order to justify changes in ICS dose in a clinical setting (Sont et al., 1999; Lipworth et al., 2012; Turton et al., 2012).

This review will discuss the BPTs available for clinical use and outline the different mechanisms by which each test identifies AHR. We will review the effects of pharmacotherapy used in the treatment of asthma that inhibits AHR, for the purposes of documenting efficacy of treatment in research or in an individual in the clinic. Where possible we will demonstrate the differences and similarities between adults and children however the majority of research cited pertains to adult asthma.

## Direct Bronchial Provocation Tests

**Figure 1:**
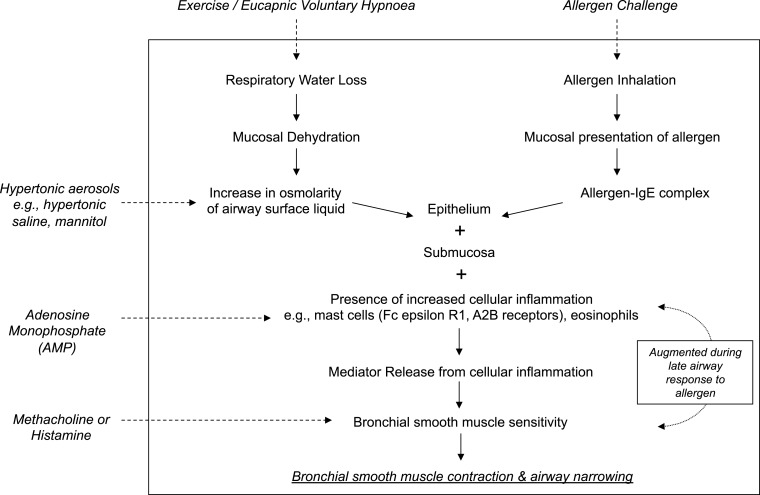
**A schematic demonstrating the mechanism of action of common bronchoconstricting stimuli delivered as standardized bronchial provocation tests in the research and clinical setting**.

Direct BPTs refer to the use of single agonist such as methacholine or histamine that act directly on receptors on the airway smooth muscle (ASM) causing contraction. The most widely used is methacholine which acts on muscarinic (M_3_) receptors, while histamine acts on H_1_ receptors (Figure 1). These agents are administered using standardized protocols either through tidal breathing using a nebulizer or deep inhalations using a dosimeter method (Ryan et al., 1981; Crapo et al., 2000), while documenting the response to each dose. A 20% fall in the forced expiratory volume in 1 s (FEV_1_) is considered as a positive test and the provocative concentration (PC_20_) or dose (PD_20_) of agonist to cause this fall is calculated by interpolation from the dose-response curve. Methacholine has regulatory approval by the United States Food and Drug Administration (US FDA) and Health Canada, however no such approvals have been obtained for histamine.

Individuals with asthma often are more sensitive and reactive to these agents compared with those who do not have asthma. It is now better understood that AHR to direct stimuli can be considered to have two components of the response that are considered “fixed” and “variable” (Cockcroft, 2010). The variable component is considered to change rapidly, for example following inhalation of an allergen which can increase AHR by acutely up-regulating airway inflammation. Alternatively, regular use of ICS, which are potent anti-inflammatory agents, is known to attenuate, though not completely inhibit AHR to direct agents (Brannan, 2010). Thus, the variable component is considered to reflect the inflammatory aspect of AHR to direct agents. Conversely, the fixed component is thought to reflect more chronic persistent (possibly permanent) structural and functional changes in the airway that may or may not be due to airway inflammation, such as airway remodeling (Cockcroft, 2010). AHR can persist despite high-dose ICS over long treatment periods (Sont et al., 1996; Brannan, 2010). The different contribution each component has on the airway sensitivity to direct stimuli cannot be determined from a single test as little research has been performed in an attempt to elucidate the components of the response. For example, it is possible that some individuals who do not have asthma may respond to direct agents (Hewitt, 2008) in the absence of an inflammatory component at the time of testing, potentially indicating the presence of a remodeling process.

Direct tests are considered to have a high diagnostic sensitivity for the presence of current asthma (Cockcroft, 2010). That is, most individuals with a clinical diagnosis of asthma will respond to these stimuli. However, several recent studies suggest that in some patient groups the diagnostic sensitivity of direct stimuli may not be as high as initially reported. The diagnostic sensitivity of methacholine in asthmatics taking regular ICS has been reported to be 77%, while the sensitivity was significantly reduced in Caucasians (69%) compared to African Americans (95%; Sumino et al., 2012). Further, there was a significant reduction in sensitivity when comparing those who were non-atopic (52%) versus atopic (82%). A similar decrease in diagnostic sensitivity of methacholine to identify asthma has been observed in non-atopic versus atopic children in cohort studies (Liem et al., 2008). Further, there is increasing evidence that AHR to direct stimuli has a low sensitivity to identify the presence of exercise-induced bronchoconstriction (EIB) in adults and children (Haby et al., 1994; Holzer et al., 2002; Anderson et al., 2009; Sue-Chu et al., 2010; Holley et al., 2012). That is, individuals with significant EIB may have no airway sensitivity to direct stimuli. However protocols that require the administration of methacholine using a deep inspiration have demonstrated decreased sensitivity to identify AHR compared to those using a tidal breathing method (Todd et al., 2004; Cockcroft and Nair, 2012). It is well established that a deep inhalation can have a protective effect on AHR in non-asthmatics (Kapsali et al., 2000). It has been hypothesized that the attenuation of the bronchodilator and/or bronchoprotective effect of a deep inspiration contribute to the severity of the clinical manifestations of asthma (Scichilone et al., 2007). However evidence for this bronchoprotection has been observed in mild asthmatics and it may be lost in the presence of more active airway inflammation and poorer lung function (Allen et al., 2008; Pyrgos et al., 2011).

Epidemiologic studies in adults and children have established that direct BPTs can have a low specificity for asthma (Woolcock and Peat, 1989). For example, it is well established that individuals with other lung disease, atopy, allergic rhinitis, and individuals who smoke may demonstrate AHR to direct stimuli (Ramsdale et al., 1985; Tashkin et al., 1992; Britton et al., 1994; Sunyer et al., 1997; Hewitt, 2008). It is not clear why these subjects respond however, it is possible that either one of or both the variable and fixed components may be present. There is evidence that reduced airway caliber may be a predictor of the response (Britton et al., 1994; Litonjua et al., 1999; Parker and McCool, 2002; Parker et al., 2003). Further, larger airways may provide some protective effect on the airway response in individuals with asthma. In a large population of physician-diagnosed asthmatics, it was observed that 27% had a negative methacholine challenge test and these subjects were more likely to have better lung function than those who had a positive test (McGrath and Fahy, 2011). Other evidence to support airway size being a determinant of AHR to direct stimuli arises from studies demonstrating that the severity of AHR decreased with age from childhood to adolescence (Sears et al., 2003). Imaging studies in humans have not provided clear evidence between a relationship with airway sensitivity to methacholine and lung function (Boulet et al., 1989).

There can be a high prevalence of AHR to direct stimuli in athletes, in particularly winter athletes who do not demonstrate significant EIB (Sue-Chu et al., 2010). This observation has supported the concept that AHR to these agents in these subjects may identify a type of airway damage or remodeling due to the effects of high intensity exercise (Kippelen et al., 2012).

In well established asthmatic populations, there is evidence of a relationship between the airway sensitivity to direct stimuli and clinically accessible markers of inflammation such as sputum eosinophils, as well as non-specific markers of inflammation such as the fraction of exhaled nitric oxide (FeNO; Jatakanon et al., 1998). However, others have not shown such relationships in patients with well established allergic asthma (Crimi et al., 1998). These differences may be accounted for by the presence of remodeling and its influence on the airway sensitivity to these agents.

## Indirect Bronchial Provocation Tests

Indirect BPTs refer to stimuli such as dry air hyperpnea or stimuli administered via an aerosol such as allergens, osmotic agents (e.g., mannitol or hypertonic saline), or adenosine monophosphate (AMP), that cause the release of a variety of mediators of bronchoconstriction from inflammatory cells (Figure 1). Mediators such as histamine, prostaglandins, and leukotrienes then act on specific receptors on the ASM to cause contraction and airway narrowing. The mast cell is thought to play a dominant role in contributing to the source of mediators (Anderson, 2010). Tryptase containing mast cells predominate in lung tissue (Andersson et al., 2009), however it is their location in the airway epithelium and the ASM that may be of significant importance in AHR (Brightling et al., 2002; Dougherty et al., 2010). There may be some involvement from airway sensory nerves, in particular to dry air hyperpnea and the osmotic stimuli as a result of airway cooling and changes in osmolarity (Anderson et al., 1996).

Inhaled allergens cause mast cell degranulation by crosslinking with Immunoglobulin E via the FcεRI receptor on the surface of the mast cell. Both dry air hyperpnea and osmotic stimuli are considered to raise the osmolarity of the airway surface, leading to movement of water into the airway lumen and a resultant increase in airway tissue osmolarity. It has been demonstrated *in vitro* that the mast cell is sensitive to osmotic change, with the ability to release histamine, prostaglandins, and leukotrienes, importantly in the presence of IgE (Eggleston et al., 1984, 1990; Gulliksson et al., 2006). AMP acts directly on mast cells to cause degranulation and release of mediators via action on the A2b receptor (Van Schoor et al., 2000).

Indirect test protocols include exercise testing either via a treadmill or cycle ergometer, eucapnic voluntary hyperventilation (EVH), nebulized hypertonic saline, inhaled dry powder mannitol, or AMP, all of which have well established and standardized protocols (Van Schoor et al., 2000; Anderson and Brannan, 2003). Inhaled mannitol has been approved by regulatory authorities in 26 countries including the US FDA as a standardized test kit (www.mannitoltest.info). Allergen inhalation is primarily used in research as it can cause a late airway response approximately 6–8 h following the early bronchoconstriction (O’Byrne et al., 2009a). However, the clinical use of specific allergen inhalation tests is limited to specialized tertiary care centers, for example to evaluate work-related asthma (Tarlo et al., 2008).

Exercise and EVH are given as a bolus dose of ventilation and changes in airway caliber are measured by the forced expiratory volume in 1 s (FEV_1_) over 15–20 min following the stimulus and comparing this to the baseline FEV_1_ value (Anderson and Brannan, 2003). A positive response is documented as a 10–15% fall in FEV_1_ and the severity of the response is determined by the degree of the fall in FEV_1_. In known asthmatics, large falls in FEV_1_ can be observed when using these tests so for clinical use they are often confined to individuals who are suspected of having EIB with normal lung function (Brannan et al., 1998; Porsbjerg and Brannan, 2010). These tests differ from the dose-response challenges such as the osmotic stimuli or AMP where, like the direct challenges, the stimulus is given in increasing doses and changes in FEV_1_ are documented until a 15% (mannitol, hypertonic saline) or 20% (AMP) fall or no target response is obtained by the maximum dose. The airway sensitivity is defined as the provoking dose of the stimulus to cause a 15 or 20% fall in FEV_1_ which is calculated by linear interpolation of the dose-response curve (PD_15_ or PD_20_). Some studies have investigated the use of a 10% fall in FEV_1_ to mannitol (PD_10_) for both monitoring ICS and identifying EIB in elite athletes (Holzer et al., 2003; Lipworth et al., 2012). These tests have a good safety profile as large falls in FEV_1_ can be avoided (Joos et al., 2003; Brannan et al., 2005).

There have been few studies firmly establishing the use of these challenge tests to investigate conditions that may mimic asthma or EIB, such as laryngeal obstruction (e.g., vocal chord dysfunction), inspiratory stridor, or other conditions such as hyperventilation syndrome in either adults or children (McFadden and Zawadski, 1996; Rundell and Spiering, 2003; Weinberger and Abu-Hasan, 2007). The presence of symptoms suggestive of asthma, in the absence of AHR to an indirect test certainly warrants further investigation. Additional investigations which may be helpful include examination of the shape of the flow-volume loop, direct laryngoscopy, and the measurement of end-tidal CO_2_ on exercise.

**Figure 2:**
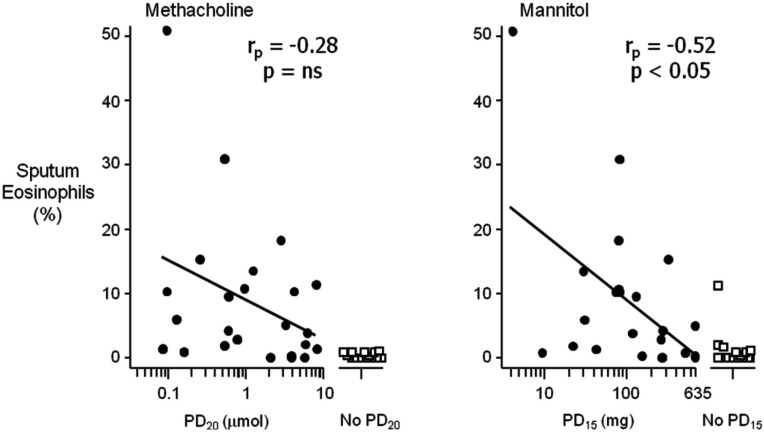
**The relationship between the airway sensitivity to inhaled methacholine and mannitol compared to the percentage (%) of eosinophils in sputum in a selected group of steroid naïve asthmatic subjects**. Airway hyperresponsiveness (AHR) to mannitol is defined as a 15% reduction in the forced expiratory volume in 1 s (FEV_1_) to a cumulative dose of less than 635 mg (PD_15_); for methacholine as a 20% reduction in FEV_1_ to less than eight micromoles (PD_20_). The relationship of % eosinophils with those who had a positive AHR (closed circles) was significant for mannitol (*r_p_* = −0.52, *p* < 0.05) compared to methacholine (*r_p_* = −0.28, *p* = ns). Those who had no AHR to either mannitol or methacholine (open squares) in this group of subjects had significantly less sputum eosinophils (adapted from Porsbjerg et al., 2008).

**Figure 3:**
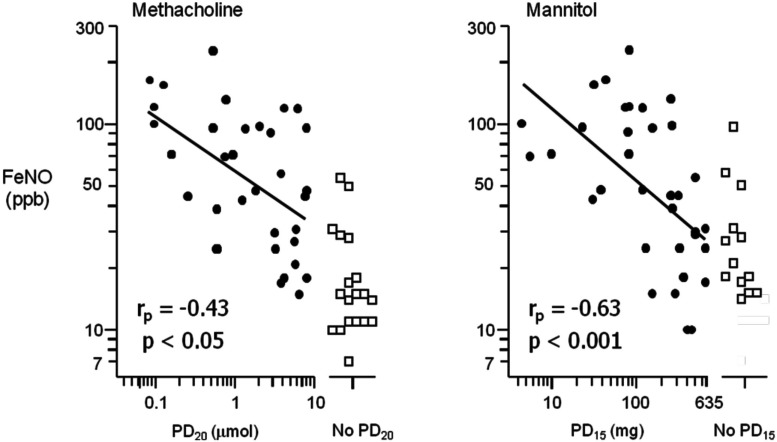
**The relationship between the airway sensitivity to inhaled methacholine and mannitol compared to the fraction of exhaled nitric oxide (FeNO) in parts per billion (ppb) in a selected group of steroid naïve asthmatic subjects**. Airway hyperresponsiveness (AHR) to mannitol is defined as a 15% reduction in the forced expiratory volume in 1 s (FEV_1_) to a cumulative dose of less than 635 mg (PD_15_); for methacholine as a 20% reduction in FEV_1_ to less than eight micromoles (PD_20_). The relationship of FeNO with those who had a positive AHR (closed circles) was more significant for mannitol (*r_p_* = −0.63, *p* < 0.001) compared to methacholine (*r_p_* = −0.43, *p* < 0.05). There were a proportion of subjects with AHR that had normal FeNO values (adapted from Porsbjerg et al., 2008).

The mechanism by which indirect stimuli identify AHR is similar to most clinically relevant stimuli that cause AHR in individuals with asthma. These stimuli require the presence of inflammation in the airway. Thus, a response to these stimuli identifies the interaction of the two key features of asthma, inflammation, and AHR. It has been well established that the airway sensitivity to indirect agents is related to the presence and degree of airway inflammation. The severity of the airway response to hypertonic saline is related to the presence of mast cells in brush biopsy and in sputum in children (Gibson et al., 1998, 2000). Those with EIB demonstrate significantly greater numbers of mast cells in the airway epithelium compared to those without EIB (Hallstrand et al., 2011). There are significantly more eosinophils in the sputum of asthmatics with EIB compared to those without EIB and there is a relationship with the presence of eosinophils in sputum and the severity of EIB (Yoshikawa et al., 1998; Kivity et al., 2000; Duong et al., 2008) and the airway sensitivity to mannitol (Porsbjerg et al., 2008). Further, studies comparing direct and indirect BPTs have demonstrated that the airway sensitivity to both AMP and mannitol have a significant relationship to the degree of eosinophils in induced sputum when compared to methacholine in the same asthmatic subjects (Van Den Berge et al., 2001; Porsbjerg et al., 2008; Figure 2). There are also studies identifying significant relationships with FeNO, a non-specific marker of airway inflammation with the airway sensitivity to exercise and mannitol (Scollo et al., 2000; Porsbjerg et al., 2008; Figure 3). However, a proportion of asthmatics who have airway sensitivity to mannitol have FeNO values well within the normal range (i.e., <20ppb; Porsbjerg et al., 2008; Cowan et al., 2010). Further, both eosinophilic and non-eosinophilic phenotypes have been observed in asthmatics responsive to both hypertonic saline and mannitol (Simpson et al., 2006; Porsbjerg et al., 2009). Non-eosinophilic phenotypes may include those with neutrophils or those who have neither neutrophils or eosinophils in sputum (also known as paucigranulocytic). These studies have shown milder AHR to indirect stimuli in subjects with a non-eosinophilic phenotype. This data suggests that there are subjects with AHR which involves active mast cells in the absence of significant eosinophilia or raised FeNO levels.

There is increasing evidence that indirect stimuli cause mast cell mediator release *in vivo*. Studies attempting to measure mediators of bronchoconstriction have identified increases in arterial plasma histamine levels measured at the time of maximal airway narrowing to exercise (Anderson et al., 1981). Leukotriene E_4_ (LTE_4_) or the metabolite of prostaglandin D_2_, 9α,11β-PGF_2_, a marker of mast cell release, are measured in urine (Reiss et al., 1997; O’Sullivan et al., 1998b; Mickleborough et al., 2003) and sputum (Hallstrand et al., 2005) following EIB. Similar observations identifying increases in urinary 9α, 11β-PGF_2_ have been made with mannitol, hypertonic saline (in children), and EVH (Brannan et al., 2003; Mai et al., 2005; Kippelen et al., 2010) as well as allergen challenge (O’Sullivan et al., 1998a), further supporting the role of the mast cell in the airway response to these stimuli. Some of these studies have also found an increase in both 9α,11β-PGF_2_ and LTE_4_ in the urine of non-asthmatics who have no BHR with indirect stimuli (Brannan et al., 2003; Mickleborough et al., 2003; Mai et al., 2005). Thus, the sensitivity of the ASM is an important component of the response to indirect stimuli.

The degree of AHR to direct stimuli is often associated with the airway sensitivity to indirect stimuli in individuals with established asthma (Anderson et al., 1997; Koh and Choi, 2002). However, airway responses to indirect stimuli can occur in the absence of AHR to either methacholine or histamine in adults and children (Haby et al., 1994; Holzer et al., 2003; Anderson et al., 2009; Holley et al., 2012). The explanation for the lack of relationship presumably lies in the differences in mechanisms of action of both direct and indirect stimuli. A variety of endogenous mediators are involved in the airway response to indirect stimuli. These include leukotrienes and prostaglandins which are both known to be more potent at causing airway narrowing than methacholine or histamine (O’Hickey et al., 1988). Thus EIB in the absence of an airway response to direct stimuli may be a result of a heightened sensitivity to endogenous mediators (Anderson, 2010).

## Assessing Efficacy of Asthma Pharmacotherapy Using AHR

### Direct AHR

Airway hyperresponsiveness to direct stimuli is used as a research tool in drug development to assess the efficacy of new and existing therapies used in the acute and long-term treatment of asthma. The ability of these therapies to inhibit AHR has in turn provided some explanation as to the mechanisms of direct AHR. Acutely, β_2_-agonists are powerful inhibitors of ASM contractility capable of decreasing AHR to direct stimuli (Page and Spina, 2006). β_2_-agonists protect against ASM contraction from direct stimuli by non-specific functional antagonism via β_2_ receptor-induced ASM relaxation. The methacholine challenge is a recognized methodology for assessing efficacy and duration of action of bronchoprotection and the pharmacoequivalence of β_2_-agonists (Parameswaran et al., 1999; O’Byrne et al., 2009b). While these drugs are the most effective pharmacotherapy to inhibit the airway response to direct stimuli, the protection is not complete in all subjects (Page and Spina, 2006). Asthmatics who regularly use β_2_-agonists can demonstrate increased airway sensitivity to direct stimuli, a decreased bronchoprotection, as well as a slower recovery to these challenge tests following a standard dose of rescue β_2_-agonist (Cheung et al., 1992; Kalra et al., 1996; Haney and Hancox, 2005). This may need to be considered when investigating the efficacy of a β_2_-agonist.

The acute use of a leukotriene antagonist and the mast cell stabilizing drugs demonstrate minimal to no effect on AHR to direct stimuli (Patel, 1984; Boner et al., 1987; Crimi et al., 1987; Davis and Cockcroft, 2005). This suggests there are limitations in using direct stimuli to investigate the bronchoprotective effect of antagonists that target other receptors or agents that stabilize the mast cell. However, long-term use of cromoglycate and montelukast has shown some small effect on attenuating the sensitivity to direct stimuli (Bel et al., 1990; Groot et al., 1992; Hakim et al., 2007). The short acting anti-cholinergic ipratropium bromide has a potent inhibitory effect on AHR to methacholine that is lost over 12 h (Crimi et al., 1992; Illamperuma et al., 2009). However the longer acting anti-cholinergic tiotropium demonstrates more potent inhibition and longer duration of protection against methacholine-induced bronchoconstriction (O’Connor et al., 1996).

**Figure 4:**
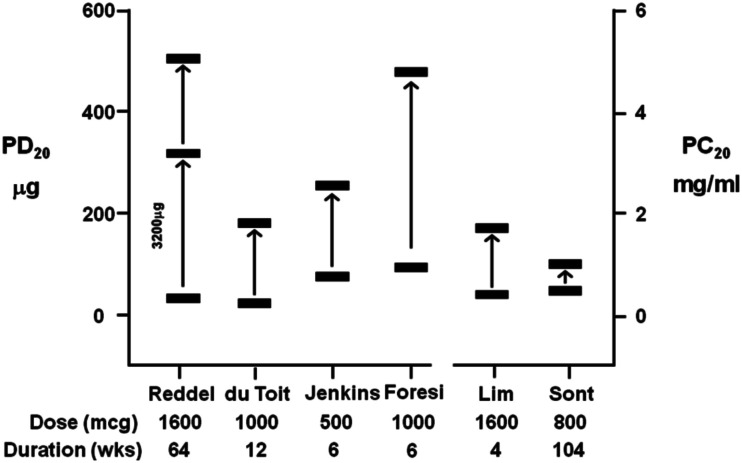
**A summary of the improvement in AHR following treatment with inhaled corticosteroids (ICS) expressed as a provoking dose (PD20) or provoking concentration (PC20) to cause a 20% fall in FEV1 from six studies using either histamine or methacholine (du Toit et al., 1997; Lim et al., 1999; Sont et al., 1999; Reddel et al., 2000; Foresi et al., 2005; Jenkins et al., 2005)**. Significant AHR to direct stimuli remains in the presence of high doses of ICS over short- and long-term treatment periods.

Regular use of ICS can attenuate direct AHR (Juniper et al., 1990; Reddel et al., 2000). AHR to direct BPTs have been used to guide the dose of ICS to monitor asthma compared to asthma guidelines in both adults and children (Sont et al., 1999; Nuijsink et al., 2007). However, AHR to direct stimuli may remain in many individuals with asthma on long-term ICS therapy over months to years of treatment (Figure 4). The short-term improvements in direct AHR as a result of ICS therapy may be related to reductions in airway inflammation (du Toit et al., 1997), while more long-term improvement in AHR may be indicative of a reduction in airway remodeling (Sont et al., 1999).

The repeated evaluation of direct AHR to aid in clinical monitoring of the efficacy of ICS was made using a treatment strategy aimed at reducing AHR to methacholine (AHR strategy) compared to a parallel group who were treated based on recommendations in the existing guidelines (reference strategy) in asthmatic adults (Sont et al., 1999). Following 2 years of ICS therapy, there was a 1.8-fold lower incidence of mild exacerbations and a significantly sustained improvement in pre-bronchodilator FEV_1_ using the AHR strategy. This was associated with improvements in the reticular layer thickness beneath the epithelium, suggesting improvements in airway remodeling. Interestingly, AHR documented as a mean PC_20_ to methacholine of 0.47 mg/mL before treatment, was not attenuated using the AHR strategy (increase in PC_20_ 1.1 doubling concentrations, not statistically significant). This confirmed previous studies showing that long-term treatment with ICS does not necessarily reduce AHR to methacholine to anywhere near the normal range (e.g., >16 mg/mL) or abolish AHR to direct stimuli (Figure 4; du Toit et al., 1997; Lim et al., 1999). A similar study in asthmatic children over 2 years found no increase in the number of symptom-free days using the AHR strategy (Nuijsink et al., 2007). They did however observe a better pre-bronchodilator FEV_1_ in a sub-group of allergic asthmatic children. To afford these clinical benefits, both studies observed the use of higher dose of ICS using the AHR strategy. While these results are inconclusive about monitoring ICS with direct stimuli these studies demonstrate it is difficult to identify optimal therapy when the AHR to these stimuli remains.

### Indirect AHR

Pharmacotherapy for the treatment of asthma has also revealed much about the mechanism of indirect AHR. Acute use of β_2_-agonists demonstrates the most potent protective effect on indirect stimuli, likely due to a combination of non-specific functional antagonism of the released mediators causing bronchoconstriction, as well as via β_2_-receptor-mediated mast cell stabilization (Anderson et al., 2006). However, regular use of β_2_-agonists, like their effects on direct stimuli, may increase the airway sensitivity to indirect stimuli, decrease the ability of β_2_-agonists when used acutely to bronchoprotect, and have decreased efficacy when being used to recover from indirect bronchoconstriction (Hancox et al., 2002; Anderson et al., 2006; Haney and Hancox, 2006). Acute use of drugs such as sodium cromoglycate and nedocromil sodium inhibits AHR to indirect stimuli due to their mast cell stabilizing properties (Brannan et al., 2000, 2006; Kelly et al., 2001; Kippelen et al., 2010). The expected increases in the urinary excretion of the mast cell marker 9α,11β-PGF_2_ following both mannitol and EVH are attenuated in the presence of cromoglycate and a β_2_-agonist (Brannan et al., 2006; Kippelen et al., 2010). The mast cell stabilizing drugs may also play a role by inhibiting airway sensory nerves or acting on the airway epithelium (Anderson et al., 1996), however they have little effect on suppressing cough to osmotic stimuli in relation to their powerful inhibitory effect on indirect AHR (Koskela et al., 2005). Leukotriene antagonists such as montelukast are powerful at inhibiting the action of leukotrienes on the ASM, causing a rapid recovery from AHR to indirect stimuli (Reiss et al., 1997; Brannan et al., 2001). Histamine antagonists have a weak though noticeable effect on reducing airway sensitivity to some indirect stimuli (Brannan et al., 2001; Anderson and Brannan, 2002; Dahlen et al., 2002). Research using these drugs has revealed the importance of the mast cell and pre-formed histamine on the initial airway response, while leukotrienes are subsequently released “de novo” to sustain bronchoconstriction to indirect stimuli. Anti-cholinergic drugs do demonstrate inhibition on indirect stimuli such as exercise and hypertonic saline (Boulet et al., 1989). However the protection is incomplete and not uniform, demonstrating wide interindividual variability. However this does not diminish the role of the neural response to indirect stimuli, as it is well known that these stimuli can activate sensory nerves which are likely responsible for cough, even in the absence of bronchoconstriction (Koskela et al., 2004, 2005). There is some evidence that interindividual variability for the bronchoprotection to exercise is related to the degree of cardiac vagal activity (Knopfli et al., 2005). However there is little data assessing the role of the newer more potent anti-cholinergics on BHR to indirect stimuli and further research is warranted to investigate the role of airway sensory nerves.

**Figure 5:**
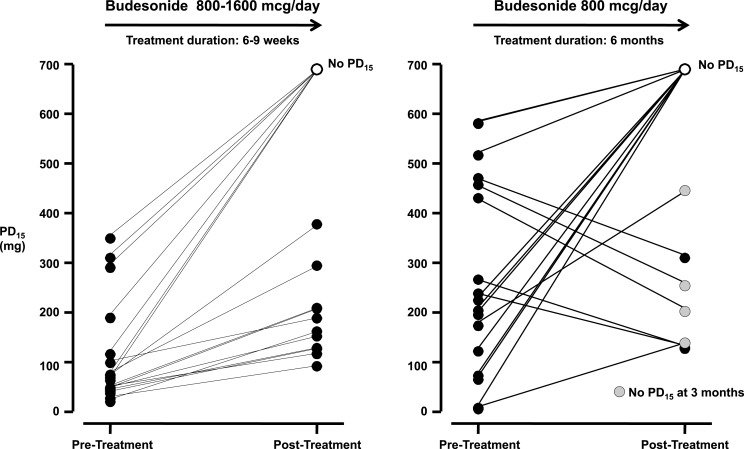
**The provoking dose of mannitol to cause a 15% fall in FEV_1_ (PD_15_) following short-term (6–9 weeks) and long-term (6 months) treatment with inhaled corticosteroids (ICS; Brannan et al., 2002; Koskela et al., 2003)**. Following 6–9 weeks 7/18 subjects had no PD_15_. Following 6 months treatment 10/17 subjects had no PD_15_ and the airway reactivity was within the non-asthmatic range, however four subjects with a PD_15_ at 6 months had no PD_15_ at 3 months. AHR in individuals on ICS who were once negative to mannitol can result following a decrease in ICS dose (Leuppi et al., 2001) and suggest these subjects may have decreased ICS adherence.

The regular use of ICS is well known to attenuate but also abolish the airway sensitivity to indirect stimuli (Koh et al., 2007). Abolishing responses to indirect stimuli using ICS may provide an objective marker for asthma control (Brannan, 2010). Such an endpoint may also indicate an opportunity for down-titration of ICS dose (Leuppi et al., 2001; Brannan et al., 2012). It is well established that both mast cells and eosinophils, known to be sensitive to ICS, play an important role in the airway response. Both cells are known to decrease in number in the presence of regular ICS therapy (Djukanovic et al., 1992). Thus, an airway response to an indirect stimuli is thought to identify an individual who is likely to have AHR that will benefit from ICS. Thus they have potential for monitoring the efficacy of ICS in both adults and children (Jonasson et al., 2000; Brannan et al., 2002; Koskela et al., 2003; Duong et al., 2008; Lipworth et al., 2012). There is also evidence of an increase in AHR to indirect stimuli in individuals with established asthma who have ICS withdrawn during down-titration of ICS (Leuppi et al., 2001). Improvements in airway sensitivity to mannitol with regular ICS occurs in association with the clinical improvements expected with ICS over weeks to months of treatment (Brannan et al., 2002; Koskela et al., 2003; Figure 5). Clinical questionnaires such the Asthma Control Questionnaire (Juniper et al., 1999b) and the Asthma Quality of Life Questionnaires (Juniper et al., 1999a) that have been validated to identify clinical improvement after weeks of ICS therapy (Juniper et al., 1994), show significant clinical improvements in AHR to mannitol following the introduction of ICS (Baraket et al., 2012; Turton et al., 2012). There are also improvements in airway reactivity with ICS which is documented as the response dose ratio (% final fall in FEV_1_ divided by the dose of mannitol to cause that fall). This is a useful outcome to document efficacy of regular treatment using ICS that results in no significant reduction in FEV_1_ (i.e., <10 or 15%). The reactivity to mannitol can be reduced in asthmatics following effective ICS therapy to levels similar to those observed in non-asthmatic subjects (Brannan et al., 2012).

A recent study assessed the use of the mannitol challenge to monitor progress to ICS by re-assessing AHR every 2 months for a year following the introduction of ciclesonide. Those who had AHR monitored had significant reductions in mild exacerbations compared to those monitored using asthma guidelines (Lipworth et al., 2012). Those who had their asthma monitored using inhaled mannitol also had greater reductions in AHR to mannitol, methacholine, and FeNO levels compared to those monitored using guidelines. This finding suggested greater improvement in both AHR and airway inflammation with the AHR strategy. This was observed in association with significant improvements the frequency of night and day symptoms and decreased β_2_-agonist use, which was not observed in those monitored using the reference strategy that used asthma guidelines. This study also showed more ICS were used in subjects managed using an AHR strategy, however this twofold increased dose of ciclesonide showed no significant increase in urinary cortisol compared to the lower doses in the reference strategy. Considering this outcome, AHR to mannitol was mild but was still present over 12 months of ICS treatment.

When using an indirect stimulus to monitor ICS, an end point to identify optimal treatment is the documentation of no AHR to these stimuli (Koskela et al., 2003; Brannan et al., 2007). In both adults and children, it has been shown that a loss in responsiveness to these stimuli is possible following weeks to months of the commencement of ICS, with more rapid benefits (over 8–12 weeks) observed in those with milder AHR (Jonasson et al., 2000; Brannan et al., 2002). The loss of AHR to these stimuli suggests that there has been a significant decrease in the cellular source of mediators following regular ICS. The inhibition of indirect AHR in those initially demonstrating airway sensitivity to these stimuli before ICS, suggests a sufficient decrease in the presence and interaction of the key features of asthma, airway inflammation and AHR. Future studies need to address the longer-term clinical benefits on outcomes such as exacerbations and asthma control in those who achieve a loss of AHR to indirect stimuli following regular ICS therapy. Such a strategy may assist in achieving the required outcome of asthma guidelines that recommend control of asthma using the minimum dose of ICS [Global Initiative for Asthma (GINA), 2007].

## Conclusion

Tests for AHR are useful objective measures to aid in asthma diagnosis by identifying a central feature of asthma. It is important to understand the differences in mechanisms between direct and indirect tests in order to employ them appropriately both in a clinical and research environment. Both direct and indirect tests for AHR have revealed much about the mechanisms of AHR in asthma, as well as the mechanisms of action of pharmacotherapy used to inhibit AHR. Future research should evaluate the features of airway remodeling that may contribute to the airway response as well as further evaluation of their role in monitoring asthma therapy such as ICS in order to achieve and sustain asthma control on minimal therapy.

## Conflict of Interest Statement

Dr. John D. Brannan was involved in the development of inhaled mannitol (Aridol^™^/Osmohale^™^) that is a registered bronchial provocation test for airway hyperresponsiveness to assist in the diagnosis of asthma. He receives a 10% portion of the Royalties paid to his prior employer, Royal Prince Alfred Hospital. He holds shares in Pharmaxis Ltd.
